# Ceftriaxone-associated dysbiosis decreases voriconazole bioavailability by upregulating intestinal P-glycoprotein expression through activation of the Nrf2-mediated signalling pathway

**DOI:** 10.3389/fphar.2024.1522271

**Published:** 2025-01-03

**Authors:** Xiaokang Wang, Chunxiao Ye, Xixiao Yang, Maoxun Yang

**Affiliations:** ^1^ The Marine Biomedical Research Institute, Guangdong Medical University, Zhanjiang, China; ^2^ Department of Pharmacy, Shenzhen Longhua District Central Hospital, Shenzhen, China; ^3^ Department of Pharmacy, The Second Affiliated Hospital of Guangzhou Medical University, Guangzhou, China; ^4^ Department of Pharmacy, Shenzhen Hospital, Southern Medical University, Shenzhen, China; ^5^ Guangdong Provincial Key Laboratory of Research and Development of Natural Drugs, Guangdong Medical University, Dongguan, China; ^6^ School of Pharmacy, Guangdong Medical University, Dongguan, China

**Keywords:** intestinal bacteria, ceftriaxone, voriconazole, bioavailability, P-glycoprotein

## Abstract

**Objectives:**

The purpose of this study was to investigate the effect of intestinal dysbiosis on the bioavailability of voriconazole and to explore any underlying mechanisms.

**Method:**

Sprague-Dawley rats were randomly divided into two groups: a normal control group and a ceftriaxone-associated dysbiotic group. The composition of the intestinal flora was examined using 16S rRNA sequencing analysis. Voriconazole concentrations were determined by high-performance liquid chromatography-tandem mass spectrometry. Outer membrane vesicles (OMVs) of microbes from the different groups were prepared for *in vitro* study in Caco-2 cells. The Nrf2 pathway and its related proteins involved in modifying P-glycoprotein (P-gp) expression were clarified by a series of immunoblot analyses.

**Key findings:**

The diversity and richness of intestinal bacteria, especially the abundance of gram-negative bacteria, were significantly decreased after ceftriaxone treatment. The AUC(0-t) and C_max_ of voriconazole were reduced, and greater voriconazole clearance were noted in the dysbiotic group. An *in vivo* study also indicated that the expression of P-glycoprotein was significantly increased after ceftriaxone treatment, which may be due to the absence of gram-negative bacteria in the intestine. Finally, *in vitro* findings in Caco-2 cells treated with OMVs from the ceftriaxone-associated dysbiotic group suggested that Nrf2 translocation into the nucleus induced high expression of P-gp.

**Conclusion:**

OMVs from intestinal bacterial in the ceftriaxone-associated dysbiotic group induced high P-gp expression by regulating the Nrf2 signalling pathway, which led to an *in vivo* reduction in the bioavailability of voriconazole due to ceftriaxone-associated dysbiosis.

## Introduction

Invasive fungal infections (IFI) are a globally emerging threat to humans (Lamoth et al., 2017). Voriconazole is a second-generation triazole that is widely used in the treatment of IFI and in antifungal prophylaxis. Its mechanism of action involves the inhibition of cytochrome P450-dependent 14 α-sterol demethylase to prevent the biotransformation of lanosterol to 14 α-demethylated lanosterol (Moriyama et al., 2017). The absolute bioavailability of voriconazole in healthy volunteers was ∼96%, whereas bioavailability was reduced to a mean of 61.8%, with a range of 44.6%–64.5% in patients. The reduction in bioavailability is thought to be due to differences in first-pass metabolism or diet, according to current reports (Shi et al., 2019). As the first–line treatment of IFI, voriconazole has saved the lives of many patients; however, many clinical investigations have revealed that the serum concentration of voriconazole exhibits remarkable variability among patient populations (Lee et al., 2021; Wang et al., 2024). For example, neutropenic patients with acute myeloid leukaemia and patients who have received an organ transplant display high variability in voriconazole bioavailability compared to normal populations (Han et al., 2010; Hicks et al., 2020; Lin et al., 2018). Previous studies have reported on the high pharmacokinetic variability of voriconazole treatment that is associated with the CYP2C19 genotype, drug interactions, hepatic dysfunction, and severe inflammation (Blanco-Dorado et al., 2020; Mafuru et al., 2019; Wang et al., 2022). However, these factors are not sufficient to explain the variability in voriconazole concentrations in patient populations (Chaudhri et al., 2020; Lee, et al., 2021).

Currently, an increasing number of studies have investigated the association between the gut microbiota and drug absorption, metabolism, efficacy and toxicity, all of which play a significant role in improving the individualized clinical use of specific drugs (Walsh et al., 2020). In recent decades, a considerable number of studies have demonstrated that the gut microbiota acts as an “invisible organ” to modulate drug pharmacokinetics (Li et al., 2020; Xun et al., 2024). The gut microbiota and the by-products it produces can metabolize some drugs into bioactive or toxic metabolites before absorption (Yoo et al., 2014). Greater than 176 drugs are known to be metabolized by at least one bacteria (Zimmermann et al., 2019). In addition, the composition and function of the gut microbiota affect the enzymes and transporters that metabolize and translocate drugs, which also play an important role in drug bioavailability (Yoo et al., 2014). For example, disorders of the intestinal flora (dysbiosis) have emerged as a new factor that affects the efficacy of statins and antiarrhythmics (Matuskova et al., 2014; Wang et al., 2018). Although the transfer system is not directly involved in drug metabolism, it can pump the drug or metabolites out of the cell. P-glycoprotein (P-gp) is an important transporter that deceases the absorption of drugs (Shi et al., 2011). Outer membrane vesicles (OMVs) produced by gram-negative bacteria prominently downregulate the expression of the P-gp transporter at the gene level (Cerveny et al., 2007; Kawauchi et al., 2014). Patients treated with voriconazole have often used antibiotics before, which easily leads to an imbalance in intestinal flora (Lahmer et al., 2021; Roberts et al., 2014). We previously found that the probiotic gut bacteria *B. fragilis* ameliorates abnormal voriconazole metabolism by inhibiting TLR4-mediated NF-κB transcription and regulating the expression of drug-metabolizing enzymes and drug transporters (Wang et al., 2021a). Nevertheless, data on the effects of dysbiosis on the bioavailability of voriconazole are lacking.

Multidrug resistance (MDR) is a major obstacle to chemotherapeutic drug therapy and is often the result of an overexpression of drug efflux proteins (such as P-gp), as a consequence of hyperactivation of nuclear factor kappa B (NF-κB) or NF-E2 p45-related factor 2 (Nrf2) transcription factors (Suttana et al., 2010). Impaired Nrf2 function is associated with drug transporters and pharmacokinetic parameters of substrate drugs (Shen and Kong, 2009). Under oxidative stress conditions, Nrf2 disassociates from its repressor Kelch-like ECH associating protein 1 (Keap1) and translocates to the nucleus, where it binds to antioxidant response elements (AREs) in the promoters of numerous genes and promotes the transcription of target genes (Hayes and Dinkova-Kostova, 2014). The degree of oxidative stress was accompanied by changes in P-gp activity. As a key antioxidant pathway, Nrf2-Keap1 pathway protects cells/organisms from the detrimental effects of oxidative stress (Chew et al., 2021). OMV proteins from clinical strains containing nonredundant proteins were identified in the membrane fractions and were associated with pathogenesis and specialized secretion systems for the delivery of virulence factors, and these proteins play a potential role in relevant processes, such as the oxidative stress response and drug kinetics (Librando et al., 2006; Mendez et al., 2012). Therefore, exploring the mechanisms of OMVs in the Nrf2 signalling pathway may be an effective method to elucidate voriconazole bioavailability in patients receiving therapy.

Our previous findings revealed that exogenous substances (xenobiotics), such as advanced oxidation protein products (AOPPs), upregulate P-gp by activating the Nrf2 signalling pathways. Thus, it is probable that these changes may modify the non-renal clearance of drugs (Xun et al., 2020). The objective of the present study was to clarify whether OMVs from gram-negative bacteria could modify the expression of P-gp and whether the Nrf2 pathway was involved in this process. We first utilized ceftriaxone-associated dysbiosis models to investigate the effect of dysbiosis on P-gp expression *in vivo*. Next, the effect of intestinal OMVs on P-gp was observed in Caco-2 cells. Finally, we highlighted the molecular mechanism by which OMVs upregulate the expression of P-gp. These findings clarify the mechanism by which the bioavailability of voriconazole varies in disease states, which helps provide new insight into the individualized clinical dosage of voriconazole in patients with IFI.

## Materials and methods

### Animals

Specific pathogen-free (SPF) Sprague–Dawley (SD) rats (male, aged 6 weeks, weighing 200–220 g) were obtained from the Laboratory Animal Center of Nanfang Hospital of Southern Medical University (Medical Experimental Animal Number: SCXK–2016–0041). All animal experimental procedures and post-treatment were approved by the Animal Ethics Committee of Nanfang Hospital (Approve No. NFYY–2020–73). Rats were housed on a 12 h light/dark cycle at a temperature of 25°C ± 2°C and humidity of 55% ± 10%. Rats with autoclaved chow and water in a pathogen-free facility. All rats were acclimated for 1 week before the experiment. Animals were euthanized 24 h after voriconazole administration, and their intestinal tract and intestinal faecal matter were separated. Immunoblot analysis and 16S rRNA-sequencing analysis were performed on tissue and faecal samples.

### Dysbiosis models

To induce dysbiosis, 10 male SPF SD rats were orally treated with ceftriaxone sodium (Roche, Beijing, China) at a dose of 2 g/kg twice a day for 8 days, as described in previous studies (Wang, et al., 2018). The remaining rats were orally administered 2 mL sterile water twice a day for 8 days as a control.

### 16S rRNA-sequencing analysis

To ensure that the model of intestinal dysbacteriosis was successfully established and to understand the differences in fecal bacterial composition between the two groups, fecal samples were collected 8 days after administration of ceftriaxone sodium or sterile water. Samples were collected within 10 min of defecation using sterile gloves or sterile spoons and were mixed thoroughly by hand in a sterile bag. A subsample was retained for analysis. Prior to DNA extraction, samples were frozen at −80°C. Samples were analyzed using 16S rRNA sequencing to assess the diversity and richness of the samples.

The DNA of the microbial community genome was obtained from faecal samples using the E.Z.N.A.^®^ DNA Kit (Omega Bio-tek, Norcross, GA, United States) per the supplier’s instructions. One percent agarose gel electrophoresis was used to detect the extraction quality of DNA, and NanoDrop 2000 (Thermo Scientific, Wilmington, United States) was used to determine the concentration and purity of DNA. The V3-V4 hypervariable region of the 16S rRNA gene was amplified by PCR using primer pairs 338F (5′-ACT​CCT​ACG​GGA​GGC​AGC​AG-3′) and 806R (5′-GGACTACHVGGGTWTCTAAT-3′). After amplification, a 2% agarose gel was used to recover PCR products. Then, AxyPrep DNA Gel Extraction Kit (Axygen Biosciences, Union City, CA, United States) was used to purify the recovered products, and a Quantus gel Fluorometer (Promega, United States) was used to quantitatively the recovered products. NEXTFLEX Rapid DNA-Seq Kits (BioScientific, Austin, TX, United States) were used to build the paired-end library. Paired-end sequencing was performed on Illumina’s MiSeq PE300/NovaSeq PE250 (Illumina Inc., San Diego, CA, United States) using standard protocols at Majorbio Bio-Pharm Technology Co. Ltd. (Shanghai, China). Raw 16S rRNA gene sequencing reads were demultiplexed, quality-filtered by fastp version 0.20.0 and merged by FLASH version 1.2.7.

### Pharmacokinetics analysis

Twenty male SD rats in one batch were randomly divided into 4 subgroups. All rats in the control group (n = 10) and dysbiosis group (n = 10) were fasted for 12 h prior to the pharmacokinetic experiment, and allowed to drink water freely. One subgroup (n = 5) in each group was used for omeprazole blood concentration for PK analysis, and the other subgroup (n = 5) was used for voriconazole blood concentration for PK analysis. The rats were orally administered with voriconazole (Vfend^®^ Tablets; Ascoli Piceno, Italy) at a dose of 25 mg/kg or omeprazole (Losec^®^ Tablets; AstraZeneca Plc., United Kingdom) at a dose of 15 mg/kg. The proportion and concentrations of voriconazole were designed according to clinical dose conversion between humans and laboratory animals (Nair and Jacob, 2016). Rat blood samples (300 μL) were collected into heparinized tubes *via* the ophthalmic veins at 0.08, 0.25, 0.5, 1, 1.5, 2, 4, 6, 8, 12, and 24 h after oral administration of voriconazole or omeprazole. 5% (w/v) CMC-Na was the solvent for oral gavage of voriconazole and omeprazole in the pharmacokinetic study. The blood samples were centrifuged at 3,000 rpm and 4°C for 10 min to isolate plasma. The plasma samples were stored at −80°C before high-performance liquid chromatography-tandem mass spectrometry (HPLC–MS/MS) analysis. The pharmacokinetic parameters were evaluated using DAS 2.0 software (Bontz Inc., Beijing, China).

### HPLC–MS/MS conditions

Concentrations of voriconazole and omeprazole were detected using a Waters ACQUITY UPLC- QTOF system (Waters, Milford, MA, United States). An ACQUITY UPLC BEH C18 1.7 µm column (Waters, Milford, MA, United States) was used, with a column temperature of 40°C. Chromatographic separation was achieved using a gradient mobile phase consisting of 0.01% formic acid in water (A) and acetonitrile (B) with a flow rate of 0.3 mL/min as follows: *voriconazole (VRC),* 5%–99% B (0–5.0 min), 99% B (5.0–7.0 min), 99%–5% B (7.0–7.1 min), and 5% B (7.1–10.0 min); *omeprazole*, 10%–85% B (0–1.5 min), 85% B (1.5–6.0 min), 85%–10% B (6.0–7.0 min), and 10% B (7.0–10.0 min). Analytes were quantified using electrospray ionization (ESI) in MS scan mode.

### Preparation of outer membrane vesicles (OMVs)

On the 7th day, fresh intestinal stool specimens were collected in groups, homogenized with 0.1 mol/L PBS (10%, w/v), centrifuged at room temperature 500 *g* for 10 min. Supernatant was collected and centrifuged at 4,500 *g* for 30 min. The microbial OMVs were then prepared as previously described (Gao et al., 2018). Briefly, supernatants were discarded and sediments were re-suspended in Luria-Bertani broth to obtain a gut microbiota suspension at 2 mg/mL. Suspensions were incubated at 37°C for 24 h. After incubation, suspensions were centrifuged at 6,000 *g* for 20 min. The supernatants were filtered by a 0.45 mm vacuum filter, and the filtrate was filtered by a 0.22 mm vacuum filter to remove the remaining cells. The filtrates were then ultracentrifuged at 200,000 *g* for 4 h at 4°C. The pellets containing the OMVs were suspended in PBS to use in cell culture experiments. A BCA Kit (Thermo ScientificTM Pierce CetM BCA Protein Assay Kit, Waltham, MA, United States) was used to determine protein concentrations.

### Cell culture and treatments

The human Caco-2 intestinal epithelial cell line was purchased from BeNa Culture Collection Biological Technology Co., Ltd. (Beijing, China) and grown in Dulbecco’s modified Eagle medium (DMEM) supplemented with 10% fetal bovine serum, 1% nonessential amino acids, streptomycin (100 U/mL), and penicillin (100 U/mL). Cells were cultured in 5% CO_2_ and 95% humidified air at 37°C in six-well plates. The culture medium was changed every 3 days until cells reached a confluent state. Caco-2 cells were then incubated in the presence or absence of the Nrf2 inhibitor ML385 (1 μmol/L) for 30 min, followed by 20 μg/mL OMVs for an additional 24 h. Cells from each group at different time points were harvested for PCR or immunoblot analysis. Each assay was repeated at least thrice.

### Cell counting kit 8 (CCK8) assay

A CCK8 (Boster, Wuahan, China) was used to examine cell viability. Cells were washed with phosphate-buffered saline (PBS). A defined number (1 × 10^4^) of cells was centrifuged after collection and resuspended in fresh solution and then 10 μL of CCK8 reagent was added to each well of the 96-well plate. The plates were incubated at 37°C for 1.5 h. The control well contained no cells but had the medium and CCK8. The absorbance values at OD 490 nm were monitored at 0, 24, and 48 h using an ELISA plate reader (Biotek, Winooski, VT, United States). Cell viability (%) = [OD (Sample)-OD (Blank)]/[OD (Control)-OD (Blank)] × 100%.

### Quantitative reverse transcription-polymerase chain reaction (qRT–PCR)

P-gp gene (ABCB1) and Nrf2 expression levels were evaluated in Caco-2 cells using qRT–PCR. Samples were homogenized and mRNA was extracted using TRIzol reagent (Invitrogen, MA, United States). mRNA concentration was determined using an ABI Prism 7,900 sequence detection system (Applied Biosystems, Foster City, CA, United States). Five hundred nanograms of total mRNA was dissolved in a 20–μL reaction system, and RNA was reverse transcribed to generate cDNA. The reaction system was as follows: 1 μL oligonucleotide (dT) primer, 4 μL 5 × reaction buffer, 1 μL RNase inhibitor, 2 μL dNTP (10 mmol/L) and 1 μL reverse transcription (RT) buffer. The mixture was denatured at 42°C/60 min and annealed at 70°C/5 min. Following RT, 4 mL of each cDNA was dissolved in 50 mL PCR mixture consisting of 26 mL 1 × SYBR Green master mix, 1 μL each primer (final concentration 0.2 mmol/L) and 18 mL sterile water. The primer sequences for the target and internal control genes GAPDH are provided in Supplementary Table S1. The amplification conditions consisted of an initial denaturation at 95°C/30 s followed by 60 cycles of 95°C/5 s, and 60°C/30 s. The fluorescence data were collected and analysed using ABI 7,500 system SDS software version 1.4 (Applied Biosystems, Foster City, CA, United States) at the end of the elongation step per cycle. Quantification was performed using the 2^−ΔΔCt^ method based on three replicates to determine the fold changes of relative abundance normalized to endogenous glyceraldehyde 3-phosphate dehydrogenase (GAPDH).

### Dual-luciferase reporter gene assay

The plasmids pGL3-ARE-luc was used to analyse Nrf2 activity. The pRL-TK Renilla luciferase (Promega, United States) plasmid was used to control for transfection efficiency. For the dual-luciferase reporter gene assay, Caco-2 cells were cultured in 10 cm cell culture dishes to 70%–80% density. A total of 0.5 μg of the pGL3-ARE-luc plasmid or the equivalent amount of control plasmid was transfected using LipofectamineTM 3,000 transfection reagent (Invitrogen, Carlsbad, CA, United States) according to the manufacturer’s protocol. At 24 h posttransfection, cells were treated with or without OMVs (20 μg/mL), and firefly and Renilla luciferase reporter signals were determined immediately using the dual-luciferase reporter gene assay system (Promega, Madison, WI, United States) (Xun, et al., 2020).

### Small interfering (si) RNA-mediated knockdown

Inhibition of Nrf2 expression in Caco-2 cells was performed using directed siRNA reagents. Nrf2-specific siRNA (siNrf2) and negative control siRNAs (siNC) served as controls and were purchased from Ribo BIO (GuangZhou, China). siRNA sequences were listed in Supplementary Table S1 siRNA was transfected into cells using Lipofectamine™ 3,000 (Thermo Fisher Scientific, Waltham, MA, United States) according to the manufacturer’s instructions. Cells were rinsed and harvested for immunoblot analysis 24–48 h after transfection.

### Western blotting analyses

All animals were sacrificed to obtain small intestine issues. Caco-2 cells (1 × 10^6^ cells/well) were placed in 6-well plates in a 5% CO_2_ incubator at 37°C overnight. The cells were then treated with lysis buffer with protease and phosphatase inhibitors from Beyotime (Institute of Biotechnology, Nantong, China) for 60 min and centrifugated at 12,000 × *g* 4°C for 10 min. The cytosolic and enriched nuclear fractions were prepared using a Nuclear Extract Kit (Active Motif, Carlsbad, CA, United States). The small intestine of each animal was washed using stroke-physiological saline solution and then homogenized with 1% protease inhibitor in RIPA buffer. After centrifugation (12,000 rpm, 12 min, 4°C), the supernatant was collected for later experiments. Protein concentration was measured using BCA kits. Ten micrograms of total protein lysate were electrophoretic in a 10% SDS-polyacrylamide gel electrophoresis (SDS–PAGE), and proteins isolated in the gel were transferred to PVDF membranes (EMD Millipore, Billerica, MA, United States). The membranes were blocked with 5% milk blocking solution at room temperature for 2 h. The total protein concentration of the supernatant was detected using a SpectraMax M5 Multifunctional enzyme marker (Molecular Devices, Sunnyvale, CA, United States). The proteins were separated by 10% SDS–PAGE, transferred to PVDF membranes and blocked for 1.5 h. Thereafter, the PVDF membranes were washed with Tris-buffered saline with Tween-20 (TBST) and incubated with primary antibodies against P-gp (1:5,000), Keap-1 (1:200), Nrf-2 (1:100) at 4°C overnight. Primary antibodies were obtained from Cell Signaling Technology (Danvers, MA, United States). After washing the membranes with TBST thrice, the membrane of each protein was incubated with secondary antibody (1:10,000, Cell Signaling Technology) for 1 h at 25°C and then washed with TBST thrice. Blots were analysis was performed with enhanced chemiluminescence (ECL) detection reagent. Images were acquired using ImageJ to quantify the optical density (Wang, et al., 2021b).

### Immunfluorescence

After treatment with OMVs alone or combined with ML385, cells were resuspended on microscopic cover slides in 35–mm Petri dishes and incubated for 24 h to allow the cells to adhere. Caco-2 cells were cultured on glass caps until 90% confluence. Cells were rinsed with PBS, fixed in 4% formalin (10 min), washed 3× with PBS, and permeabilized with 0.5% Triton X-100 (in PBS, 5 min). Then, the cells were blocked with 2% bovine serum albumin (BSA, Roche, Switzerland) for 30 min and fixed with 4% formaldehyde solution at room temperature. After that, the cells were incubated with rabbit polyclonal anti-P-gp antibody (1:100, Abcam, United Kingdom) overnight at 4°C and with fluorescein isothiocyanate (FITC) conjugated Affini-Pure goat anti–rat IgG (1:10,000; Protein-tech Biotechnology Co., Ltd., Wuhan, China) for 1 h at 37°C. Caco-2 cells were observed and photographed using a fluorescence microscope (Olympus BX-51, Olympus, Tokyo, Japan) following DAPI staining (1:1,000 in PBS, Sigma–Aldrich, St Louis, MO, United States) to visualize the nuclei.

### Statistical analyses

Experimental data are presented as the mean ± standard deviation (SD). The data were analyzed using GraphPad Prism 8.0. A Student’s t-test was used to compare the differences between two groups, and the differences among multiple groups were evaluated using the Kruskal-Wallis test which analyzes non-normally distributed, non-parametric data, followed by Dunn’s *post hoc* test. Other statistical methods were reported accordingly. *P* < 0.05 was considered statistically significant.

## Results

### Voriconazole bioavailability was reduced in ceftriaxone-associated dysbiosis

**FIGURE 1:**
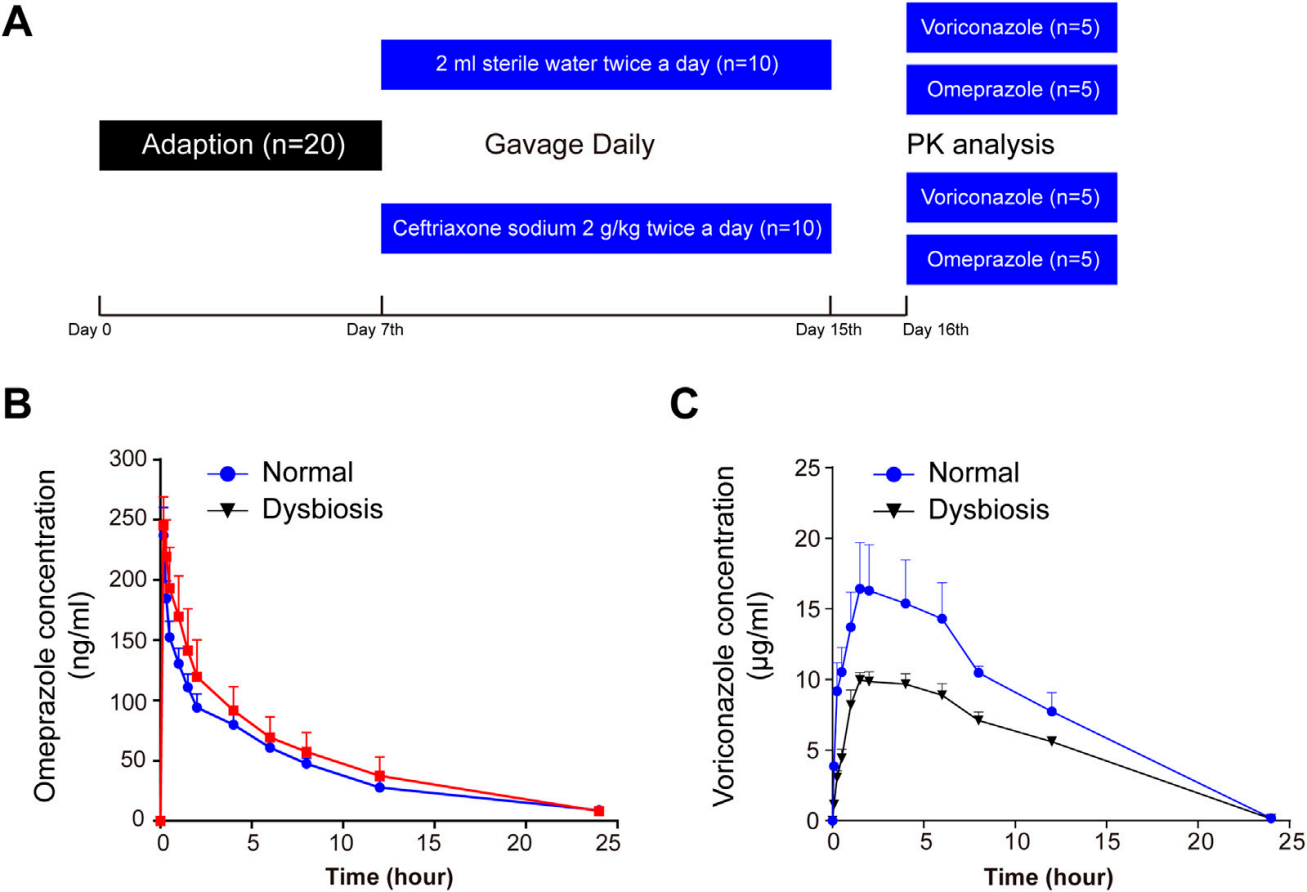
Serial monitoring of omeprazole and voriconazole (VRC) in the peripheral blood of rats. **(A)** Schematic diagram of the experimental protocol. **(B)** Blood concentration-time curves after omeprazole (15 mg/kg) treatment. **(C)** Blood concentration-time curves after VRC (25 mg/kg) treatment. Time refers to hours after oral omeprazole or VRC administration, and horizontal lines depict the mean at each time point. Error bars represent the standard deviation.

To examine whether ceftriaxone-associated dysbiosis affected the pharmacokinetics of voriconazole, we induced a model of intestinal dysbiosis in rats (Figure 1A). The model was validated using 16S rRNA sequencing analysis. CYP2C19 activity was investigated by analysing the pharmacokinetics of omeprazole in rats pre-treated with ceftriaxone, as shown in Figure 1B and Supplementary Table S2. No significant differences in CYP2C19 activity were observed between the ceftriaxone treatment group and the normal control group. These results indicated that ceftriaxone-associated dysbiosis did not affect rat hepatic CYP2C19 activity *in vivo*.

To investigate whether the alteration of intestinal flora affected pharmacokinetics in rats, blood concentrations of voriconazole were measured at 0.08, 0.25, 0.5, 1, 1.5, 2, 4, 6, 8, 12 and 24 h after oral administration of voriconazole (Figure 1C). The pharmacokinetics of voriconazole in both groups are shown in Supplementary Table S3. The AUC_(0-t)_ and C_max_ of voriconazole was decreased significantly in plasma exhibiting dysbiosis compared to normal plasma (*P* < 0.01 and *P* < 0.05, respectively). Meanwhile, the clearance of voriconazole was markedly increased in the dysbiotic group (*P* < 0.05) compared to the control group. The T_max_ and t_1/2_ of voriconazole did not differ between the dysbiotic group and the normal group. These results indicate that alterations of the intestinal microflora affect the absorption of voriconazole.

### Dysbiosis induced by ceftriaxone alters intestinal transporter expression

**FIGURE 2:**
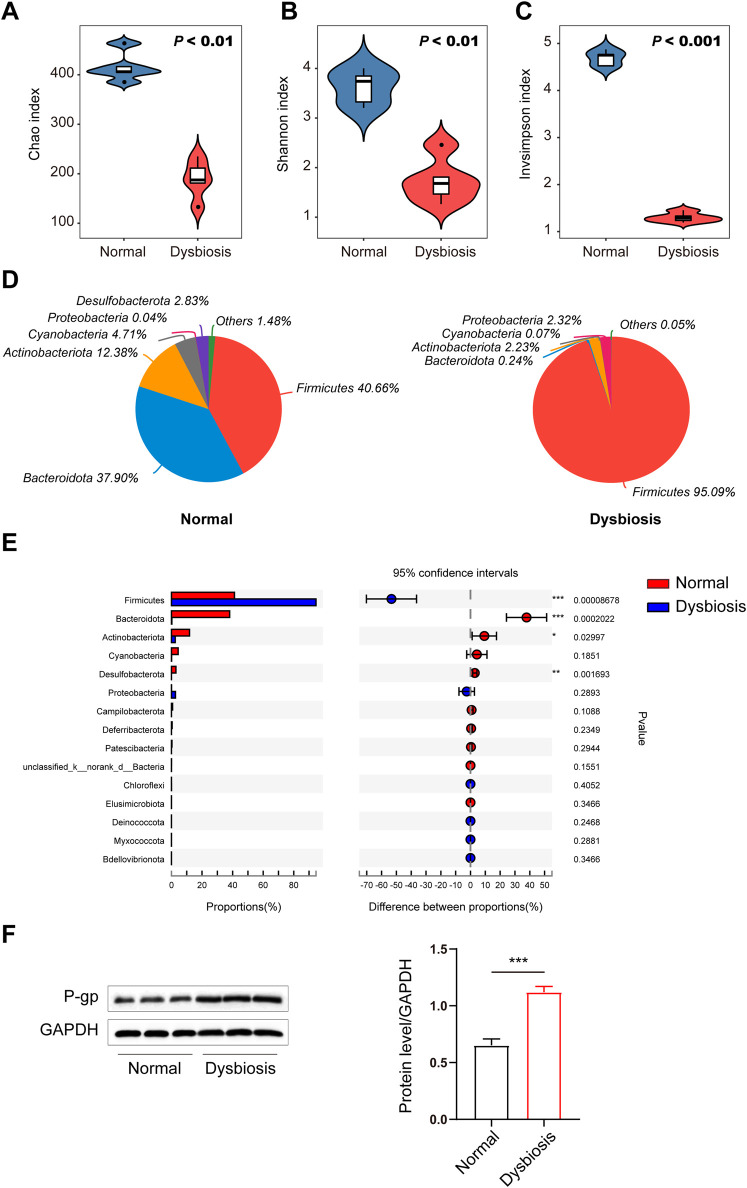
Differences in the intestinal microbiota (diversity and composition) are associated with drug bioavailability after ceftriaxone administration. **(A–C)** Gut microbial alterations in groups are expressed by alpha diversity. Community richness of the intestinal microbiota in dysbiosis is expressed as Chao index **(A)** and microbiota diversity in dysbiosis is expressed as the Shannon index **(B)** and the inverse Simpson index **(C)** compared to the normal control group (n = 6, respectively). **(D)** Community analysis pieplot at the phylum level. **(E)** Wilcoxon rank-sum test bar plot at the phylum level. **(F)** Immunoblots (left) and densitometric analysis (right) of P-gp. Immunoblots are representative of at least three independent experiments. A Student’s *t*-test was used to test for differences in alpha diversity analysis between groups. *P* values between the normal and dysbiotic groups are shown in the panels. *P < 0.05, **P < 0.01 and ***P < 0.001 vs. the normal/dysbiotic group.

We next explored the potential factors affecting the differences in drug bioavailability. We hypothesize that the gut microbiota may be an important modulator of the decreased bioavailability of voriconazole in the host and to test this we analysed the gut microbiota between normal and dysbiotic rats to understand if the gut microbiota contributed to the decrease in voriconazole bioavailability following ceftriaxone treatment. The 16S diversity analysis revealed a significant difference in alpha diversity between the two groups of rats (Figures 2A–C). The alpha diversity Chao (Figure 2A), Shannon (Figure 2B) and InvSimpson (Figure 2C) indices were significantly decreased in the dysbiotic group compared to the normal group (*P* < 0.01, *P* < 0.01 and *P* < 0.001, respectively). These observations demonstrated that ceftriaxone treatment significantly decreased the diversity and richness of the intestinal microflora in the dysbiotic group compared to the normal group. Similarly, principal component analysis of beta diversity also indicated that the microbial community was markedly different between the normal group and dysbiotic group (Figure 2D).

Relative abundance of *Firmicutes* at the phylum level increased sharply, whereas that of *Bacteroidota*, *Actinobacteriota* and *Desulfobacterota* decreased significantly in the dysbiotic group based on the effects of antibiotic treatment (ceftriaxone) against gram-negative bacteria (Figure 2E). The proportions of *Actinobacteriota* and *Desulfobacterota* decreased significantly in the dysbiotic group. In addition, multidrug resistance protein 1 (MDR1) protein efflux pump (P-gp) levels in the intestine were increased in the ceftriaxone-treated group (Figure 2F).

### Bacterial outer membrane vesicles from ceftriaxone-induced dysbiotic rats promote intestinal P-gp expression

To uncover the mechanism involved in the *in vivo* results, we conducted a mechanistic study using Caco-2 cells and a CCK-8 assay to evaluate the potential effects of different concentrations of OMVs on Caco-2 cell viability and to determine the appropriate concentration of OMVs to further study the mechanism of OMVs in Caco-2 cells.

**FIGURE 3:**
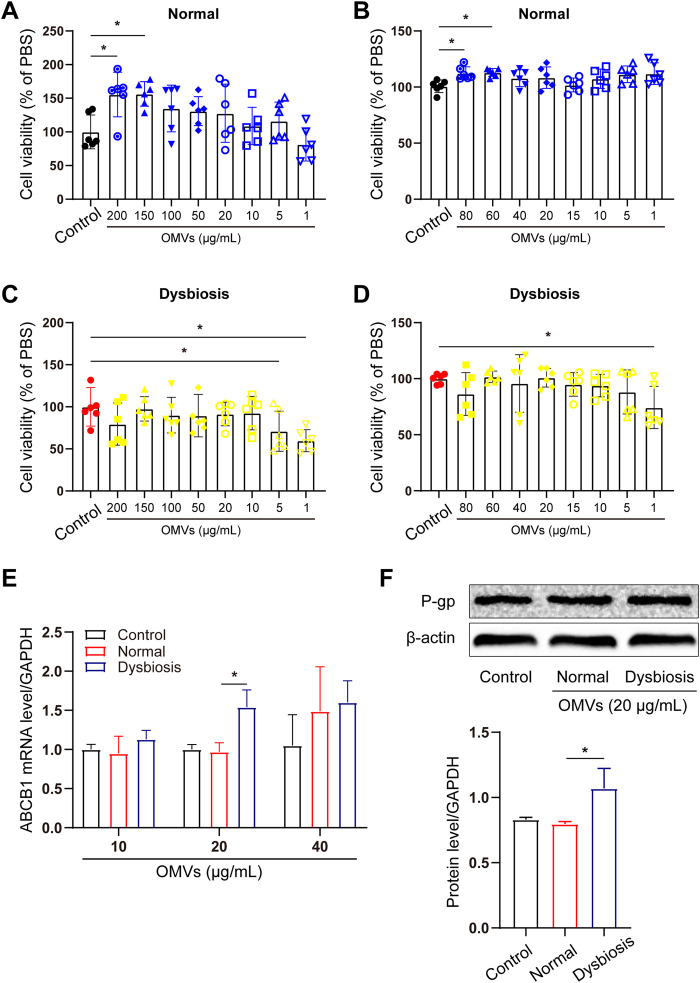
Screening of the effect of different concentration of microbial OMVs on Caco-2 cells. **(A–D)** A screening assay was performed at different OMV concentrations in Caco-2 cells. Cell viability in Caco-2 cells treated with OMVs originating from normal animals **(A, B)**. Cell viability in Caco-2 cells treated with OMVs originating from dysbiotic animals **(C, D)**. **(E)** Optimum OMV concentration selection based on P-gp gene (ABCB1) expression levels. **(F)** Western blot of P-gp in CaCo2 cells after different treatments. *P < 0.05 vs. the control/normal group.

Caco-2 cells were incubated with OMVs from normal or dysbiotic rats at different concentrations (0, 1, 5, 10, 20, 50, 100, 200 μg/mL) for 24 h. As shown in Figures 3A, C, high concentrations of OMVs (200 and 150 μg/mL) from normal rats or low concentrations of OMVs (1 and 5 μg/mL) from dysbiotic rats exhibited significant reductions in cell viability (*P* < 0.05).

To determine the optimal dosage and treatment time for OMV experiments, Caco-2 cells were incubated for 24 h with OMVs at a lower concentration (0, 1, 5, 10, 20, 40, 60, 80 μg/mL). As shown in Figures 3B, D, OMVs from normal rats at 60 and 80 μg/mL significantly increased cell viability (*P* < 0.05) while OMVs from dysbiotic rats at 1 μg/mL significantly decreased cell viability (*P* < 0.05). These results were used to further study the effects of OMVs on P-gp gene and protein levels. As shown in Figures 3E, F, Caco-2 cells were treated with OMVs at 10, 20 and 40 μg/mL for 24 h. Importantly, a significant change in ABCB1 mRNA levels were observed after OMV (20 μg/mL) exposure from both normal and dysbiotic rats (Figure 3E). Immunoblotting showed that Caco-2s cultured with 20 μg/mL OMVs derived from dysbiotic rats yielded an increase in the amount of P-gp protein (Figure 3F), which is consistent with a significant increase in ABCB1 mRNA (*P* < 0.05). This finding suggests that the modulation of intestinal efflux transporter expression was most pronounced when OMVs were present at a concentration of 20 μg/mL, a finding that facilitates further mechanistic exploration.

### OMVs upregulate efflux transporter expression by activating the Nrf2 pathway in Caco-2 cells

**FIGURE 4:**
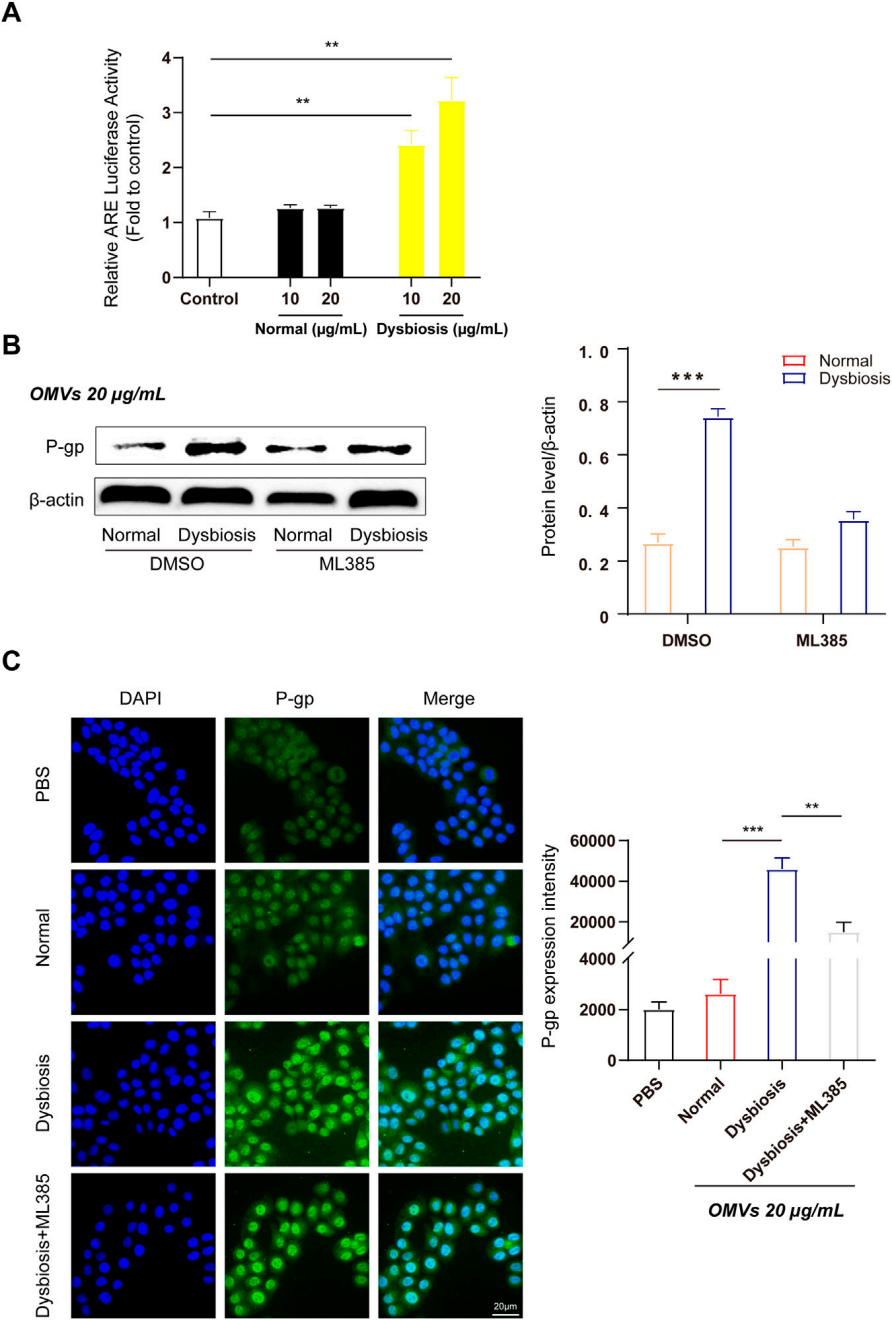
OMVs upregulate efflux transporter expression through activation of the Nrf2-mediated signalling pathway in Caco-2 cells. **(A)** OMVs from dysbiotic rats induce ARE-luciferase activity. The dual-luciferase reporter system was used to detect Nrf2-ARE signalling pathway activation in Caco-2 cells. **(B)** Left, Western blot analysis of P-gp protein expression following ML385 (1 μmol/L) treatment in normal/dysbiotic OMV-treated Caco-2 cells. Right, densitometric analysis of the proteins. **(C)** Immunofluorescence assays (left) showed the relative abundance of P-gp in various treated Caco-2 cells (scale bar = 20 μm) and quantitative analysis (right) of the protein expression from the four groups. Data are presented as the mean ± standard deviation. **P < 0.01, ***P < 0.001.

Our previous studies have shown that antioxidant response elements (AREs) are located in the promoters of numerous genes and promote the transcription of target genes (such as Nrf2). Nrf2 is a critical nuclear transcription factor involved in regulating the expression of efflux transporters and also functions as a responder to external stimuli after exposure to xenobiotic substances. We assessed the effects of OMVs on Nrf2 activity using a dual-luciferase reporter gene assay. Figure 4A indicates that Nrf2 activity was increased in a dose-dependent manner after 24 h of treatment with OMVs from dysbiotic rats, and showed a significant increase compared to the control rats (*P* < 0.01). Thus, OMVs from dysbiotic rats may act as an efficient Nrf2 inducer compared to OMVs from normal rats.

To verify the efficiency of OMVs from rats with dysbiosis, Western blotting was used to detect the protein expression of P-gp in Caco-2 cells incubated with or without ML385. As expected, quantitative analysis revealed that cells treated with OMVs from rats with dysbiosis treated but not subject to ML385 pretreatment showed a significant increase in P-gp protein levels. However, P-gp protein expression was suppressed when cells were pretreated with ML385 prior to treatment with OMVs from dysbiotic rats (Figure 4B). Further fluorescence quantification confirmed that the mean fluorescence intensity increased significantly after incubation with OMVs from rats with dysbiosis (Figure 4C, *P* < 0.001). In the ML385 pretreatment group, the mean fluorescence intensity in Caco-2 cells incubated with 20 μg/mL OMVs decreased significantly (Figure 4C, *P* < 0.01). Altogether, these findings indicate that the Nrf2 pathway may play a critical role in regulating the expression of efflux transporters.

**FIGURE 5:**
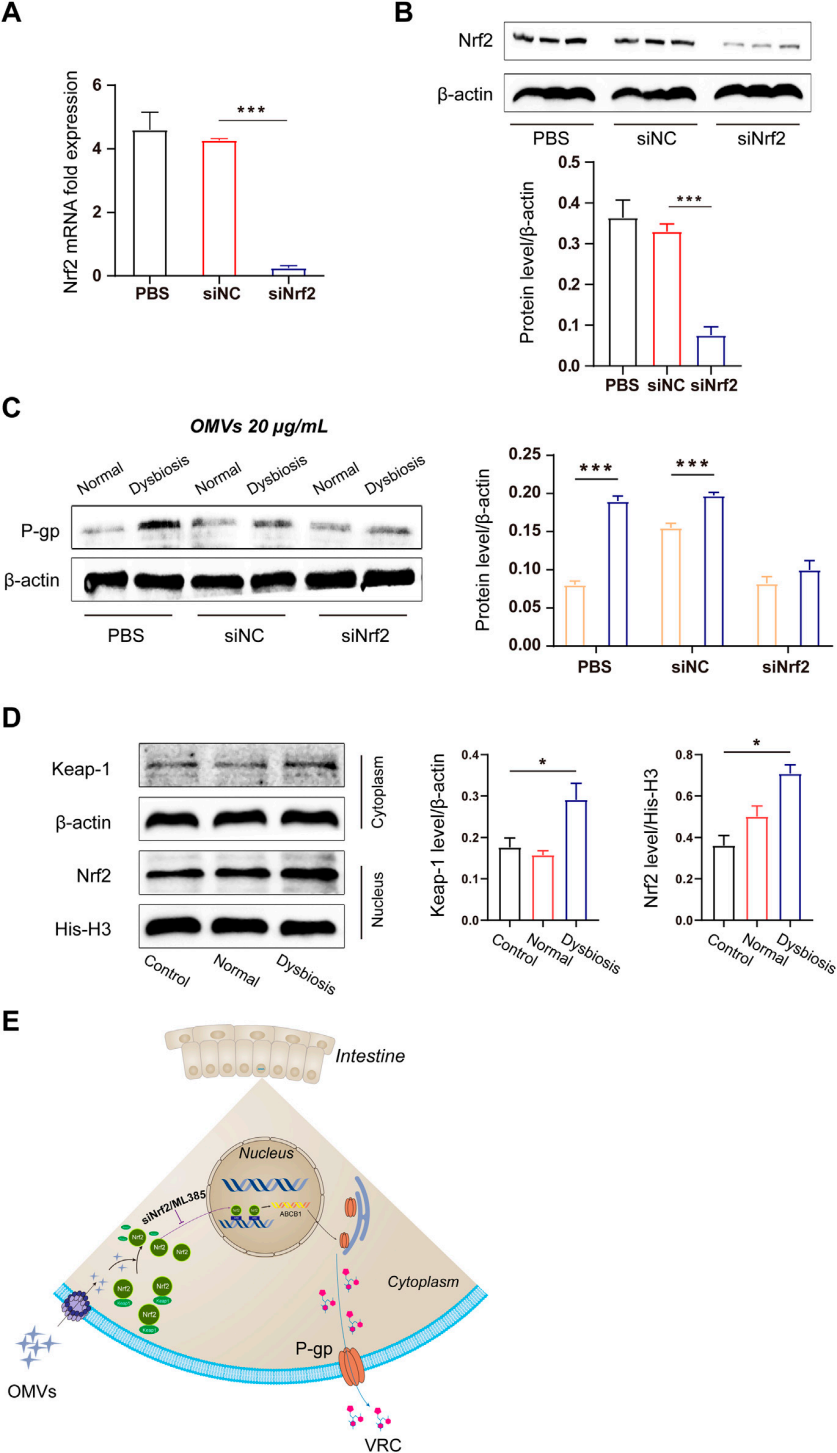
Molecular mechanism of OMV-induced efflux transporter expression. **(A)** Quantitative analysis of Nrf2 mRNA expression after small interfering RNA-mediated knockdown in Caco-2 cells. **(B)** Immunoblots and densitometric analysis of Nrf2 to verify the efficacy of Nrf2 knockdown in Caco-2 cells. **(C)** Immunoblots and densitometric analysis of P-gp expression after OMV treatment. **(D)** Immunoblots and densitometric analysis of the Nrf2-Keap1 pathway in Caco-2 cells after OMV treatment. **(E)** Schematic illustration of the OMV regulatory mechanism. Data are presented as the mean ± standard deviation. ***P < 0.001.

To further explore the mechanism by which OMVs promote the overexpression of efflux transporters, we used siRNA to knock down Nrf2 gene expression. Genetic silencing of Nrf2 led to significantly decreased levels of Nrf2 mRNA and protein in Caco-2 cells (Figures 5A, B). Additionally, and consistent with the results *in vivo*, OMVs originating from the dysbiotic group upregulated P-gp protein expression. However, this outcome was reversed by Nrf2 knockdown. Thus, this result further clarifies that Nrf2 plays a key role in the high expression of P-gp in the dysbiotic state (Figure 5C). Furthermore, we found that OMVs from rats with dysbiosis activated the Nrf2-Keap1 pathway in Caco-2 cells, as evidenced by the increased Keap1 expression in the cytoplasm and Nrf2 expression in the nucleus following OMV treatment (Figure 5D). This finding indicates that Nrf2 markedly accumulated in the nucleus after Nrf2 escaped from Keap1 in the cytoplasm (*P* < 0.05).

To further explore the detailed mechanism by which OMVs promote the overexpression of efflux transporters, we performed siRNA to knock down Nrf2 gene expression. Genetic silencing of Nrf2 led to significantly decreased Nrf2 mRNA and protein levels in Caco-2 cells (Figures 5A, B). Additionally, consistent with the results *in vivo*, OMVs (20 μg/mL) originating from the dysbiosis group upregulated P-gp protein expression. However, this outcome was reversed by Nrf2 knockdown. Thus, this result further clarifies that Nrf2 plays a key role in the high expression of P-gp in the dysbiosis state (Figure 5C). Furthermore, we found that OMVs from rats with dysbiosis activated the Nrf2-Keap1 pathway in Caco-2 cells, as evidenced by the increased Keap1 expression in the cytoplasm and Nrf2 expression in the nucleus following OMV treatment (Figure 5D). This finding indicates that Nrf2 markedly accumulated in the nucleus upon Nrf2 escape from Keap1 in the cytoplasm (*P* < 0.05).

## Discussion

A reduction in the diversity of the intestinal microbiota has been linked to several disorders, including obesity and inflammatory bowel disease (IBD) (Turnbaugh et al., 2008; Willing et al., 2010). The gut microbiota can affect drug pharmacokinetics through several mechanisms, including modulation of drug transport and the biotransformation of drugs into active or inactive metabolites. Thus, dysbiosis of the intestinal microbiota may alter the absorption of drugs, thereby altering their therapeutic efficacy and safety (Wang and Zhou, 2024). In this study, we demonstrated that the bioavailability of voriconazole was altered by the intestinal flora of rats treated with the antibiotic ceftriaxone.

The main bacteria inhabiting the intestinal tract belong to four dominant phyla: *Firmicutes*, *Bacteroidetes*, *Proteobacteria*, and *Actinobacteria*. Antibiotics are common factors that have an obvious effect on the proportions of these bacterial groups. In particular, broad-spectrum antibiotics reduce bacterial diversity while expanding and declining specific indigenous taxa (Modi et al., 2014). Analysis of the rat faecal microbiota α-diversity in our study indicated that the administration of ceftriaxone ignificantly decreased the richness of the gut microbiota. Specifically, ceftriaxone treatment reduced the abundance of *Bacteroidetes* and increased the abundance of *Firmicutes* and *Proteobacteria* at the phylum level. In our study, we found that outer membrane vesicles (OMVs) derived from these gram-negative bacteria can promote the expression of P-gp in Caco-2 cells through the Nrf2-Keap1 pathway, providing novel information that may be useful clinically in individualizing treatment plans in patients being treated with voriconazole.

Ceftriaxone is not catabolically metabolized *in vivo*, and no studies reported to date has suggested that ceftriaxone interferes with the expression of metabolism-related proteins in intestinal cell efflux transporters (Zhu et al., 2021). To examine whether ceftriaxone could affect drug metabolism, we established a model of intestinal dysbiosis in rats through the oral administration of ceftriaxone for 8 days. Ceftriaxone is reported to be a significant broad-spectrum antibiotic that induces an imbalance in the gut microbiota by widely inhibiting gram-negative bacteria (Wang, et al., 2018; Zhao et al., 2013). We verified the difference in intestinal bacteria α-diversity in the model between control and treated rats. In addition, rats were gavaged with voriconazole on the 10th day after ceftriaxone gavage, demonstrating that ceftriaxone sodium has minimal direct effects on voriconazole’s pharmacokinetics. Moreover, to avoid the influence of CYP2C19 metabolic enzymes on voriconazole metabolism in rats, we used a CYP2C19 probe substrate (omeprazole), performed a pharmacokinetic analysis, and found no difference in pharmacokinetics between the two groups. Thus, we were confident of the reliability of our dysbiosis models to explore any differences in voriconazole bioavailability in our study. After a single dose of voriconazole (25 mg/kg), plasma samples were collected from each rat and the concentration of voriconazole was measured. The AUC_(0-t)_ and C_max_ of voriconazole decreased drastically, whereas CLz/F increased significantly in the dysbiotic group compared to the control group. This finding suggests that reduced voriconazole exposure may be due to gut dysbiosis.

Voriconazole has a narrow therapeutic index and large intra/interindividual pharmacokinetics (PK) variability (Wang et al., 2021b). Previous studies have explained the inter/intraindividual variability of voriconazole in healthy populations on gene polymorphisms. However, in disease states, disease conditions, or drug-drug interactions (DDI) in different patients were thought to be responsible for the inter/intraindividual variability of voriconazole treatment, however the underlying mechanism was unknown (Kadam and Van Den Anker, 2016). When administered orally, voriconazole is mainly absorbed in the intestine, and the intestinal microbiota play important roles in governing the PK of the drug (Zhang et al., 2018). However, with the wide use of antibiotics, an imbalance of intestinal bacteria is common (dysbiosis), which affects the pharmacokinetics of drugs. Our study reveals that intestinal dysbiosis suppresses the absorption of voriconazole by increasing P-gp expression.

P-gp plays an important role in the intestinal absorption of drugs. Our study revealed that in the dysbiotic group, P-gp protein expression in the intestine was significantly increased compared to the control group, indicating that dysbiosis may decrease the bioavailability of voriconazole by increasing P-gp expression. Gram-negative bacteria interact with the host by producing secretory nanosized outer membrane vesicles (OMVs) to deliver both soluble and insoluble components into host cells (Ellis and Kuehn, 2010). LPS is one of the most abundant components of OMVs secreted by gram-negative bacteria and is considered the main promoter of the pathogenic activity of OMVs (Beutler and Rietschel, 2003). LPS is one of the most abundant components of OMVs secreted by gram-negative bacteria and is considered the main promoter of the pathogenic activity of OMVs (Beutler and Rietschel, 2003). LPS is involved in the regulation of cellular transcription factors, thereby inhibiting the active expression of enzymes involved in drug metabolism along with cellular drug transporters (Morgan et al., 2008). OMVs mediate the cytosolic localization of LPS (Vanaja et al., 2016). Indeed, previous research has indicated that OMVs produced by gram-negative bacteria are able to decrease the expressions of P-gp mRNA in Caco-2 cells (Gao et al., 2018). In our study, *Firmicutes* represented greater than 95% of the total bacteria in the dysbiotic group, indicating that less than 5% gram-negative bacteria existed in the intestinal bacteria of the dysbiotic group. In contrast, in the control group, *Firmicutes* and *Bacteroidota* accounted for 40.66% and 37.90% of the total bacteria, respectively. This result indicates that the proportion of gram-negative bacteria in the control group was more than 7-fold greater than that of the dysbiotic group. Given the significantly reduced number of gram-negative bacteria, P-gp expression was increased in the dysbiotic group compared to the control group. Thus, we hypothesized that the gut microbiota in the dysbiotic group could upregulate intestinal P-gp partially by reducing OMVs produced by gram-negative bacteria. However, the bioavailability of voriconazole may also affected by the CYP3A4, and our study did not examine any changes to this metabolic enzyme. Future studies, will continue to examine factors that alter the bioavailability of voriconazole via CYP3A4 in disease states (like an LPS-inflammatory state).

Another major finding of our study was that Nrf2 plays a critical role in regulating the expression of efflux transporters in response to gram-negative bacteria in the absence of OMV exposure. After exposure to OMVs from the dysbiotic group, Nrf2 protein expression increased, as did cytoplasmic Keap1 and nuclear Nrf-2 protein levels. These results suggest that OMVs promote Nrf2 translocation from the cytoplasm to nucleus. In order to investigate whether OMVs induce Nrf2 expression through interactions with AREs, Caco-2 cells were transiently transfected with pGL3-ARE-luc (Figure 4A). Our data showed that ARE-luciferin activity was strikingly activated by OMVs (from the dysbiotic group) at doses of 10 and 20 μg/mL. ML385 is a small molecule inhibitor of Nrf2 that specifically and directly interacts with Nrf2 proteins and blocks the transcriptional activity of Nrf2 into the nucleus. Treatment with ML385 abolished the ability of OMVs from the dysbiotic group to induce higher levels of P-gp expression (Figure 4B). The results showed that OMVs from the dysbiotic group could also upregulate the expression of nuclear Nrf2. Gene knockdown using siRNA targeted at Nrf2 further confirmed the ability of OMVs from the dysbiotic group to upregulate the expression of P-gp (Figure 5C). Taken together, these findings indicate that OMVs upregulate intestinal P-gp through Nrf2 transcriptional activation. Figure 5E shows the overall mechanism by which OMVs upregulate intestinal P-glycoprotein, based on the findings of our study.

## Conclusion

The results of our study indicate that intestinal dysbiosis markedly reduced the absorption of voriconazole in SD rats. The potential mechanism by which this occurs is related to increased intestinal expression of P-gp, through the transcriptional activation of Nrf2 by OMVs produced by microbes in the dysbiotic group. These findings may provide new insight to clarify the mechanism underlying the variability in voriconazole treatment.

## Data Availability

The 16S rDNA data presented in the study are deposited in the NCBI repository, accession number: PRJNA1189179.
