# Supplementary material for: Ceftriaxone-associated dysbiosis decreases voriconazole bioavailability by upregulating intestinal P-glycoprotein expression through activation of the Nrf2-mediated signalling pathway
Source: Front Pharmacol. 2025 Jan 3;15:1522271. doi: 10.3389/fphar.2024.1522271 (PMC11738772; doi:10.3389/fphar.2024.1522271)
Supplement: Supplementary file 1 [file Table1.docx]

**Supplementary Tables**

**Table S1** Primers sequences used in described studies

| Gene | Forward primer (5′–3′) | Reverse primer (5′–3′) |
| --- | --- | --- |
| *hABCB1* | CCCATCATTGCAATAGCAGG | GTTCAAACTTCTGCTCCTGA |
| *hNrf2* | GCACATCCAGACAGACACCA | TCAGGGGTGGTGAAGACTGA |
| *hGAPDH* | ATCCCATCACCATCTTCCAG | CCATCACGCCACAGTTTCC |
| *siNrf2* | GCCUUACUCUCCCAGUGAATT | UUCACUGGGAGAGUAAGGCTT |
| *siNC* | UUCUCCGAACGUGUCACGUTT | ACGUGACACGUUCGGAGAATT |

**Table S2** Pharmacokinetic parameters after the oral administration of omeprazole (15 mg/kg) to rats in the Normal and Dysbiosis groups.

| Parameters | Units | Normal | Dysbiosis |
| --- | --- | --- | --- |
| AUC(0-t) | mg/L h | 1017.533±188.787 | 1047.537±188.98 |
| AUC(0-∞) | mg/L h | 1109.636±233.62 | 1136.202±229.284 |
| MRT(0-t) | h | 6.587±0.628 | 6.291±0.641 |
| MRT(0-∞) | h | 8.77±1.628 | 8.166±1.773 |
| t1/2 | h | 6.722±1.301 | 6.556±1.159 |
| Tmax | h | 0.17±0.02 | 0.18±0.03 |
| CL/F | L/h/kg | 0.014±0.003 | 0.015±0.002 |
| Cmax | mg/L | 220.322±13.081 | 228.649±11.972 |

Data are presented as the mean ± standard deviation, n = 5.

**Table S3** Pharmacokinetic parameters after the oral administration of voriconazole (25 mg/kg) to rats in the Normal and Dysbiosis groups.

| Parameters | Units | Normal | Dysbiosis |
| --- | --- | --- | --- |
| AUC(0-t) | ng/mL h | 195.4 ± 14.71 | 127.8 ± 18.16^**^ |
| AUC(0-∞) | ng/mL h | 247.5 ± 93.89 | 145.0 ± 39.16 |
| MRT(0-t) | h | 7.06 ± 0.56 | 7.43 ± 0.18 |
| MRT(0-∞) | h | 6.35 ± 0.98 | 6.98 ± 0.78 |
| t1/2 | h | 8.105 ± 9.55 | 5.99 ± 3.97 |
| Tmax | h | 1.60 ± 0.22 | 1.80 ± 0.27 |
| CL/F | L/kg | 0.10 ± 0.03 | 0.19 ± 0.05* |
| Cmax | ng/mL | 16.42 ± 3.26 | 10.01 ± 1.21^*^ |

Data are presented as the mean ± standard deviation, n = 5. **P* < 0.05 and ***P* < 0.01 versus Normal group.
