# Supplementary material for: Ceftriaxone-associated dysbiosis decreases voriconazole bioavailability by upregulating intestinal P-glycoprotein expression through activation of the Nrf2-mediated signalling pathway
Source: Front Pharmacol. 2025 Jan 3;15:1522271. doi: 10.3389/fphar.2024.1522271 (PMC11738772; doi:10.3389/fphar.2024.1522271)
Supplement: Supplementary file 2 [file Presentation1.pptx]

## Slide 1
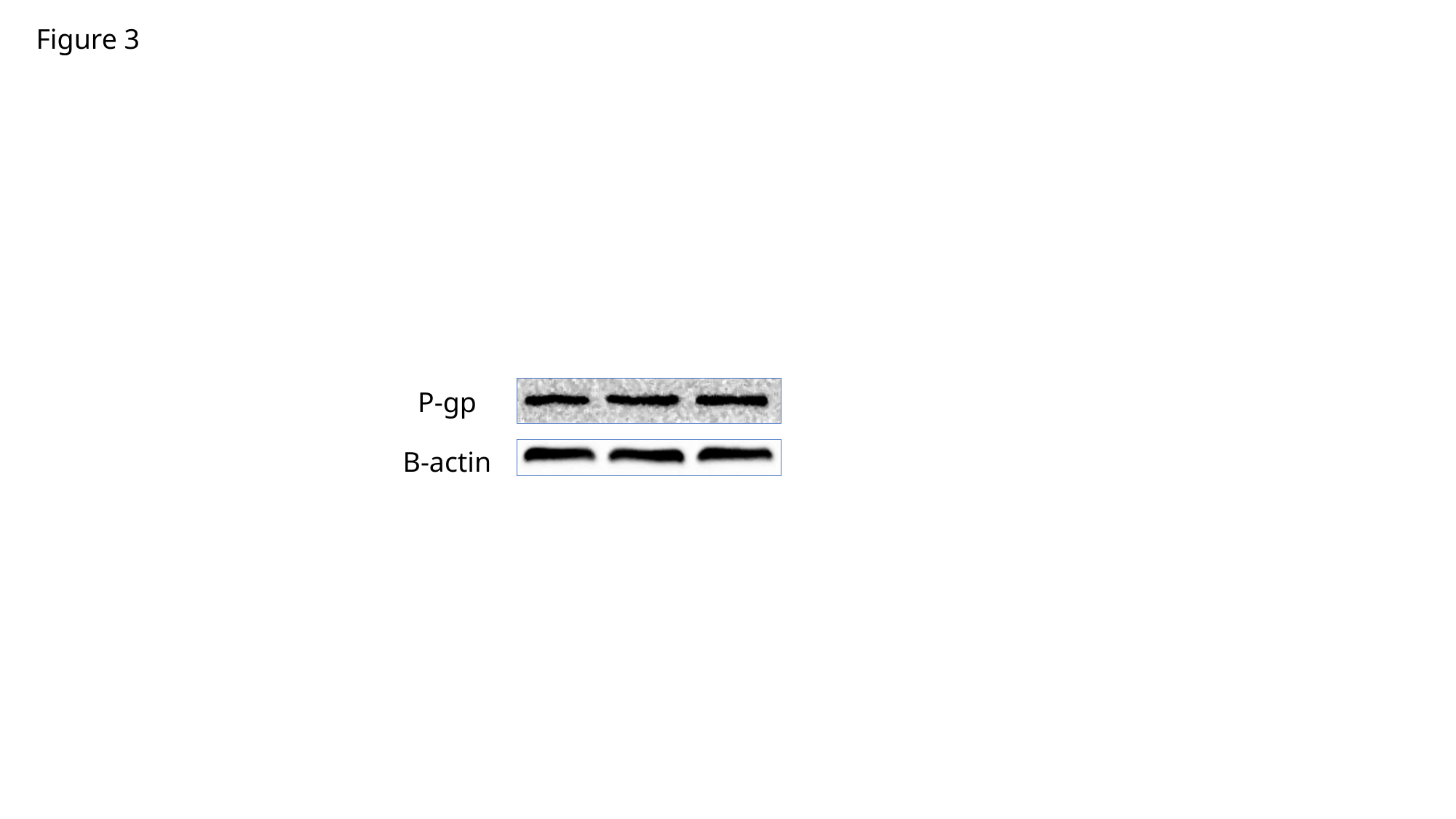

Figure 3
P-gp
B-actin

## Slide 2
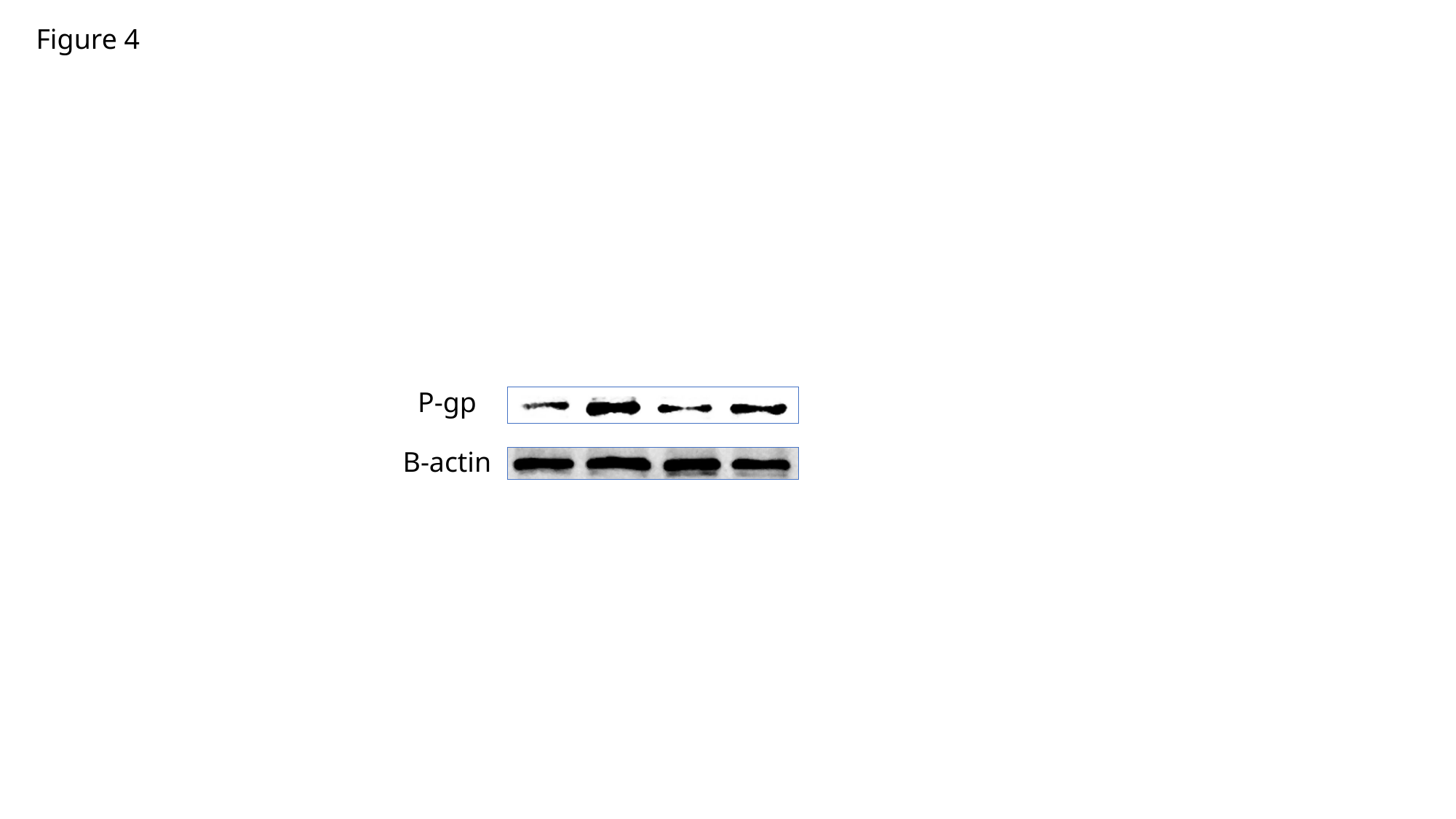

Figure 4
P-gp
B-actin

## Slide 3
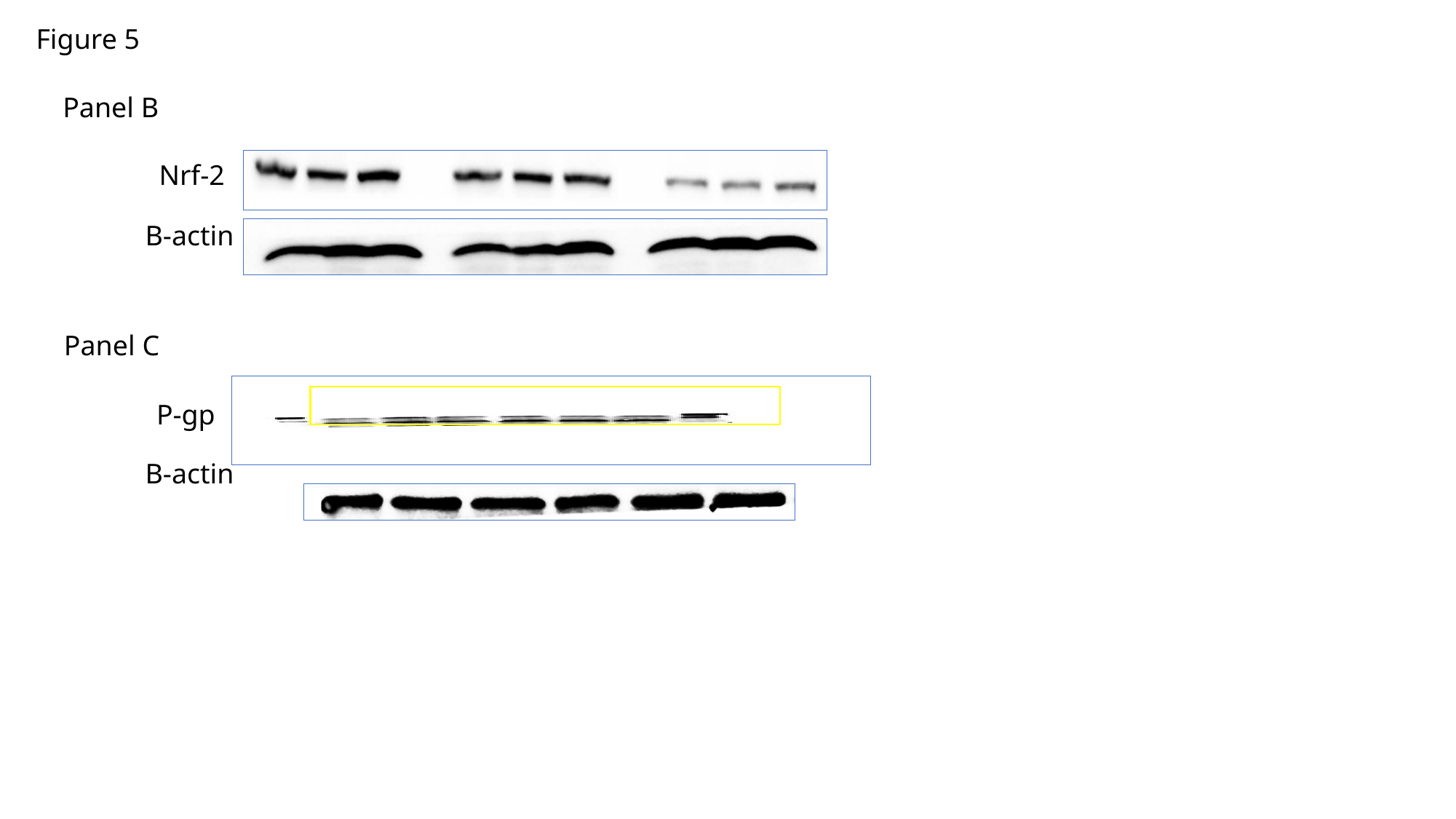

Figure 5
Panel B
Nrf-2
B-actin
Panel C
P-gp
B-actin

## Slide 4
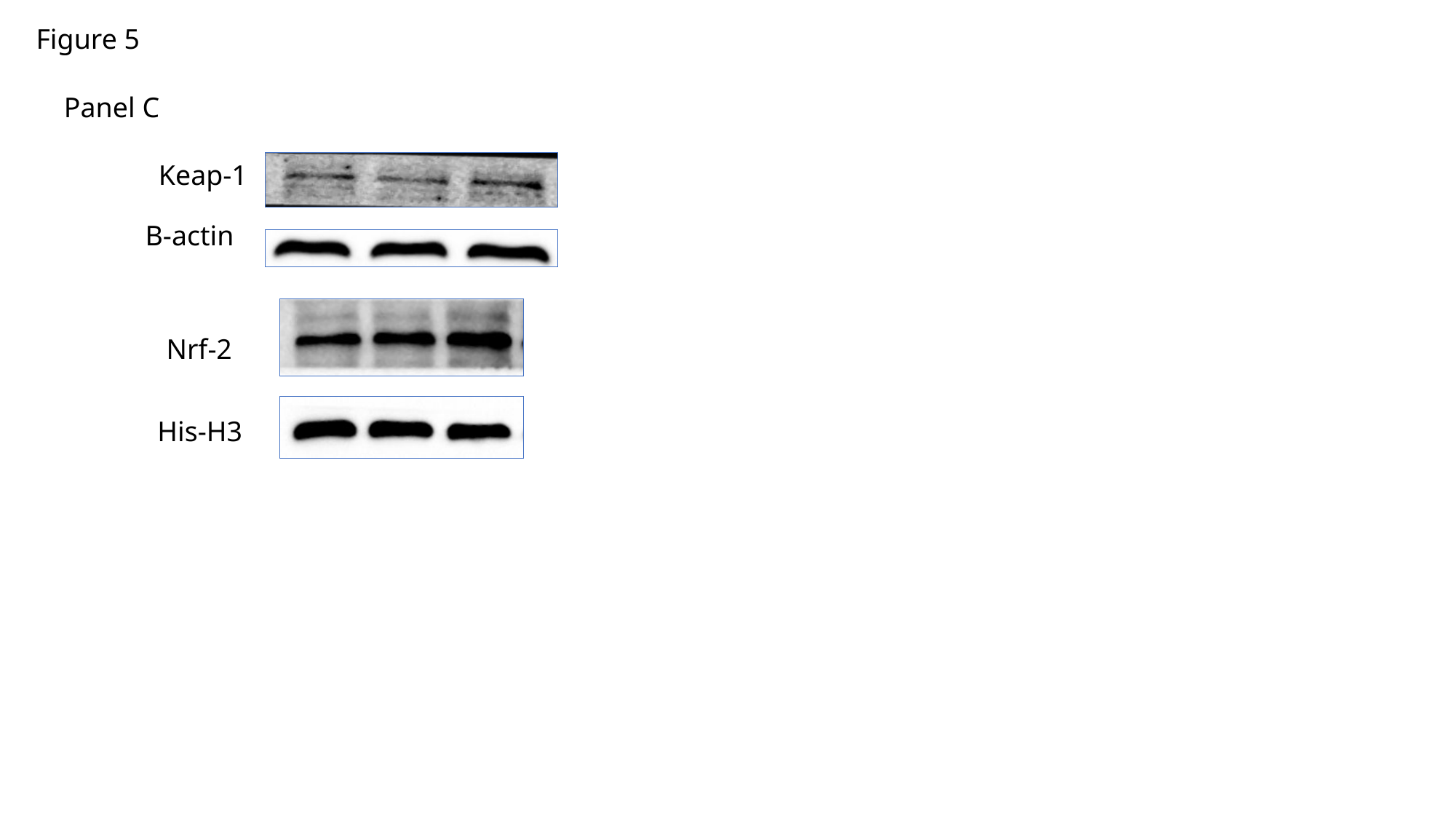

Figure 5
Panel C
Keap-1
B-actin
Nrf-2
His-H3
